# The Age-Driven Decline in Neutrophil Function Contributes to the Reduced Efficacy of the Pneumococcal Conjugate Vaccine in Old Hosts

**DOI:** 10.3389/fcimb.2022.849224

**Published:** 2022-03-23

**Authors:** Shaunna R. Simmons, Essi Y. I. Tchalla, Manmeet Bhalla, Elsa N. Bou Ghanem

**Affiliations:** Department of Microbiology and Immunology, University at Buffalo School of Medicine, Buffalo, NY, United States

**Keywords:** neutrophils, vaccines, *S. pneumoniae*, immunosenescence, antimicrobial activity

## Abstract

Despite the availability of vaccines, *Streptococcus pneumoniae* (pneumococcus) remains a serious cause of infections in the elderly. The efficacy of anti-pneumococcal vaccines declines with age. While age-driven changes in antibody responses are well defined, less is known about the role of innate immune cells such as polymorphonuclear leukocytes (PMNs) in the reduced vaccine protection seen in aging. Here we explored the role of PMNs in protection against *S. pneumoniae* in vaccinated hosts. We found that depletion of PMNs in pneumococcal conjugate vaccine (PCV) treated young mice prior to pulmonary challenge with *S. pneumoniae* resulted in dramatic loss of host protection against infection. Immunization boosted the ability of PMNs to kill *S. pneumoniae* and this was dependent on bacterial opsonization by antibodies. Bacterial opsonization with immune sera increased several PMN anti-microbial activities including bacterial uptake, degranulation and ROS production. As expected, PCV failed to protect old mice against *S. pneumoniae*. In probing the role of PMNs in this impaired protection, we found that aging was accompanied by an intrinsic decline in PMN function. PMNs from old mice failed to effectively kill *S. pneumoniae* even when the bacteria were opsonized with immune sera from young controls. In exploring mechanisms, we found that PMNs from old mice produced less of the antimicrobial peptide CRAMP and failed to efficiently kill engulfed pneumococci. Importantly, adoptive transfer of PMNs from young mice reversed the susceptibility of vaccinated old mice to pneumococcal infection. Overall, this study demonstrates that the age-driven decline in PMN function impairs vaccine-mediated protection against *Streptococcus pneumoniae*.

## Introduction

*Streptococcus pneumoniae* (pneumococcus) infections are responsible for an estimated 1.6 million deaths globally each year (WHO., 2014). Pneumococcal infections occur most frequently in young children and older adults (Grudzinska et al., 2020). In fact, *S. pneumoniae* remain the leading cause of community acquired bacterial pneumonia in people over the age of 65 years old (CDC., 2018 2018; Grudzinska et al., 2020). This is despite the availability of two pneumococcal vaccines, the pneumococcal polysaccharide vaccine (PPSV) which is recommended for older adults and the pneumococcal conjugate vaccine (PCV) which is recommended for the most vulnerable elderly in the USA (Matanock A et al., 2019). However, both vaccines have shown reduced protection in elderly individuals (Simell et al., 2011; Gonçalves et al., 2016; Matanock A et al., 2019). This is in part, due to immunosenescence, defined as the age-related decline in immune system function (Simell et al., 2011), that leads to reduced antibody levels and function following vaccination (Simell et al., 2011; Jackson et al., 2013).

Polymorphonuclear leukocytes (PMNs) are innate immune cells required for host defense against *S. pneumoniae* infection. These cells are the first to influx to the site of pneumococcal infection and are essential for bacterial clearance (Hahn et al., 2011; Kolaczkowska and Kubes, 2013; Bou Ghanem et al., 2015; Gonçalves et al., 2016). PMNs can kill bacteria through several antimicrobial effector functions. PMNs kill extracellular bacteria *via* the release of reactive oxygen species (ROS), neutrophil extracellular traps (NETs), and by degranulation of preformed granules that contain antimicrobial compounds (Kolaczkowska and Kubes, 2013; Naegelen et al., 2015; El-Benna et al., 2016; Nguyen et al., 2017; Yin and Heit, 2018). PMNs also engulf *S. pneumoniae* through the process of phagocytosis (Kolaczkowska and Kubes, 2013). Once engulfed, the bacteria are contained within phagosomes where they are killed intracellularly through ROS production, acidification of the phagosome, and primarily by fusion of antimicrobial granules with the phagosome membrane (Nordenfelt and Tapper, 2011; Monfregola et al., 2012; Johnson and Criss, 2013; Yin and Heit, 2018). The importance of these cells in bacterial killing and defense of naïve hosts against pneumococcal infection is well established (Garvy and Harmsen, 1996; Hahn et al., 2011; Bou Ghanem et al., 2015). However, whether these innate immune cells also play a critical role in protection of vaccinated hosts against *S. pneumoniae* challenge is not fully explored.

Despite different methods of bacterial killing utilized by PMNs, *S. pneumoniae* express several factors to evade PMN-mediated killing, one of which is the expression of a polysaccharide capsule (Shenoy and Orihuela, 2016). This capsule helps the bacteria resist phagocytic killing (Hyams et al., 2010). The presence of an opsonin, in the form of complement or antibody, deposited on the surface of the bacteria helps overcome this resistance and mediates clearance through activation of complement and Fc receptors on PMNs (Kadioglu et al., 2008; Shenoy and Orihuela, 2016). Activation of these receptors triggers distinct signaling pathways in PMNs (García-García and Rosales, 2002; Kobayashi et al., 2002; Nguyen et al., 2017). Previous studies using coated beads found that signaling *via* Fc and complement receptors resulted in differences in phagocytosis, ROS production as well as receptor specific changes in gene expression (García-García and Rosales, 2002; Kobayashi et al., 2002). Thus, in a vaccinated host, antibodies may enhance host protection against infection by binding pneumococci and promoting their uptake and killing by PMNs (Dalia and Weiser, 2011).

Aging is accompanied by a decline in levels and opsonic capacity of antibodies (Simell et al., 2011; Jackson et al., 2013; Adler et al., 2017) in response to immunization, which blunts the effectiveness of vaccines in protecting the host against infection. However, there is also impaired intrinsic PMN function in elderly subjects (Simell et al., 2011). When compared to young donors, PMNs from elderly human donors display reduced killing of *S. pneumoniae* even when the bacteria were opsonized with sera from young PPSV immunized hosts (Simell et al., 2011). This suggests that antibody-mediated responses by PMNs are impaired with age. Similar studies, however, are lacking for PCV. Further, the contribution of PMNs to the age-driven decline in vaccine protectiveness is unclear.

In this study, using a murine model of infection, we asked if the decline in PMN function contributes to the reduced efficacy of the pneumococcal conjugate vaccine in aged hosts. We found that following PCV vaccination, PMNs are necessary for protection of young hosts against infection with *S. pneumoniae*. Aged mice were not protected by PCV vaccination. This was associated with an age-related intrinsic decline in PMN function, specifically in intracellular killing of engulfed bacteria following antibody-mediated uptake. Importantly, adoptive transfer of PMNs from young hosts into vaccinated, aged mice, rescued their ability to fight infection. These findings indicate that enhancing PMN function in aged hosts, may boost overall vaccine protectiveness.

## Materials and Methods

### Ethics Statement

All animal studies were performed in accordance with the recommendations in the Guide for the Care and Use of Laboratory Animals. Procedures were reviewed and approved by the University at Buffalo Institutional Animal Care and Use Committee (approval number AR202100089).

### Mice

**Figure 1:**
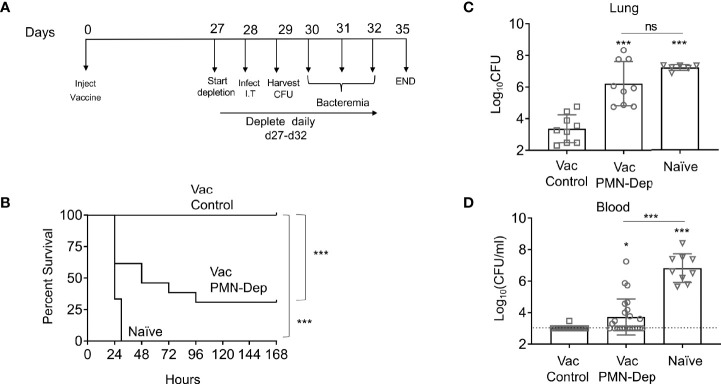
PMNs are required for protection of PCV immunized young hosts at the time of bacterial challenge. Young C57BL/6 female mice were mock treated (naïve) or administered 50μl of Prevnar-13 *via* intramuscular injections to the hind legs (vaccinated). Four weeks following vaccination mice were challenged i.t. with 1x10^7^ CFU *S. pneumoniae* TIGR4. To deplete PMNs prior to infection, mice were injected i.p. with anti-Ly6G antibodies (IA8) or isotype control at days -1, 0, and +1 to +4 with respect to infection as outlined in **(A)**. **(B)** Mice were then monitored for survival. **(B)** Data were pooled from three experiments with 14 mice per group. Significant differences were determined using the Log-Rank (Mantel-Cox) test. Bacterial burden in the lung **(C)** and blood **(D)** were also assessed 1 day post infection. **(C, D)** Data were pooled from three separate experiments with n = 9 mice per group. Each square indicates an individual mouse. *, denotes significant differences by One-way ANOVA followed by Tukey’s multiple comparisons test. * denotes p < 0.05, and *** denotes p < 0.001. ns denotes not significant.

Young (2 months) and old (18-22 months) C57BL/6 mice were obtained from the National Institute on Aging colonies or purchased from Jackson Laboratories (Bar Harbor, ME). All mice were housed in a specific-pathogen free facility at the University at Buffalo for four weeks prior to starting experiments. Due to mice availability, all experiments were performed in male mice, however the role of PMNs in vaccinated hosts was also confirmed in young female C57BL/6 mice (**Figure 1**).

### Bacteria

Wildtype (WT) *Streptococcus pneumoniae* TIGR4 strain and a pneumolysin deletion mutant (*Δply*) were a kind gift from Andrew Camilli (Greene et al., 2015). GFP-expressing *Streptococcus pneumoniae* TIGR4 was a kind gift from Sarah Roggensack (Bhalla et al., 2020). Bacteria were grown to mid-exponential phase at 37°C at 5% CO_2_ in Todd Hewitt broth supplemented with 0.5% yeast extract and oxyrase as previously described (Siwapornchai et al., 2020).

### PMN Isolation

Bone marrow cells were collected by flushing the femurs and tibias harvested from uninfected mice with RPMI supplemented with 10% FBS and 2mM EDTA. Red blood cells were then lysed, and remaining cells were washed, and resuspended in PBS. PMNs were separated *via* density centrifugation using histopaque 1119 and 1077 (Sigma) as previously described (Swamydas and Lionakis, 2013; Bhalla et al., 2020; Siwapornchai et al., 2020; Tchalla et al., 2020). The PMNs were then resuspended at the desired concentration in Hank’s Balanced Salt Solution/0.1% gelatin with no Ca^2+^ or Mg^2+^. The purity of PMNs was confirmed using flow cytometry and 85-90% of enriched cells were positive for Ly6G and CD11b.

### Mouse Infections

Mice were anesthetized with isoflurane and infected intratracheally (i.t.) with 50ul of the indicated concentrations of WT *Streptococcus pneumoniae* TIGR4 pipetted directly into the trachea with the tongue pulled out to ensure delivery of bacteria directly into the lungs. Following infection, mice were monitored for signs of disease including activity, weight loss, posture, breathing, and blindly scored from 0 (healthy) to 21 (severe disease) as previously described (Tchalla et al., 2020). Lungs were harvested and homogenized in sterile PBS. Blood was collected from the tail vein to follow bacteremia. Samples were diluted in sterile PBS and plated on blood agar plates to enumerate bacterial numbers.

### Generation of Immune Sera

To generate immune sera, mice were immunized intramuscularly *via* injection of 50ul of the pneumococcal conjugate vaccine (PCV) Prevnar-13^®^ (Wyeth pharmaceuticals) into the caudal thigh muscle. Mice were euthanized four weeks following vaccination and blood was collected by cardiac puncture into microtainer tubes (BD) and centrifuged at 9000 rpm to collect sera. Sera were stored at -80°C until use. To heat inactivate the sera, samples were incubated at 56°C for 40 minutes as previously described (Fante et al., 2021).

### Opsonophagocytic Killing Assay

The ability of PMNs to kill *S. pneumoniae* was determined as previously described (Lysenko et al., 2007; Bou Ghanem et al., 2015). 10^5^ PMNs were infected with 2x10^5^ CFU (at a multiplicity of infection (MOI) of 2) of *S. pneumoniae* TIGR4 pre-opsonized with 3% naïve, immune, or heat inactivated (Hi) immune sera as indicated. PMNs were infected for 40 minutes at 37°C, reactions were then stopped on ice for 2 minutes. Reactions were then plated on blood agar and killing percentage was determined with respect to no PMN control wells under the same treatment conditions.

### MPO and CRAMP ELISAs

PMNs were infected at a MOI of 2 with *S. pneumoniae* TIGR4 pre-opsonized with 3% naïve or heat inactivated immune sera. Control wells were mock infected with 3% sera only. PMNs were infected for 40 minutes at 37°C. Cells were then centrifuged to collect the supernatants and pellets. Pellets were lysed with RIPA lysis buffer with 0.1% Tx-100 and both, supernatants and pellets were analyzed for MPO (MPO ELISA, Invitrogen) and the Cathelicidin antimicrobial peptide (CAMP ELISA kit, mybiosource) as per manufacturer’s instructions.

### Measurement of ROS

Intracellular and extracellular ROS production was measured as previously described (Siwapornchai et al., 2020). PMNs were re-suspended in HBSS (Ca^2+^ and Mg^2+^ free) and acclimated at room temperature for one hour. The cells were then spun down and re-suspended in KRP buffer (Phosphate buffered saline with 5mM glucose, 1mM CaCl_2_ and 1mM MgSO_4_) and equilibrated at room temperature for 30 minutes. 5x10^5^ PMNs were then seeded per well in 96-well white LUMITRAC™ plates (Greiner Bio-One). Wells were infected with TIGR4 *S. pneumoniae* pre-opsonized with naïve or heat inactivated immune sera. Control wells were mock infected with PBS and 3% sera only. Phorbol 12-myristate 13-acetate (PMA) (Sigma) (100nM) was used as a positive control. For detection of extracellular ROS, 50μM Isoluminol (Sigma) plus 10U/ml HRP (Sigma) were added to the wells and for detection of intracellular ROS, 50μM Luminol (Sigma) was added to the wells as previously described (Dahlgren et al., 1989; Dahlgren and Karlsson, 1999; Martner et al., 2008; Rajecky et al., 2012). Luminescence was immediately read (following infection) over a period of one hour at 37°C in a pre-warmed Biotek Plate reader. Wells containing buffer and Isoluminol plus HRP or Luminol alone were used as blanks.

### Gentamicin Protection Assay

PMNs were infected at MOI 25 with *Δply S. pneumoniae* pre-opsonized with 3% naïve or heat inactivated immune sera for 15 minutes at 37°C. Gentamicin was then added at 100µg/ml for 30 minutes to kill the extracellular bacteria. To determine initial bacterial uptake, reactions were washed with HBSS and resuspended in HBSS/0.1% gelatin, reactions were then diluted and plated on blood agar. To determine intracellular killing, reactions continued at 37°C for an additional 30 minutes. The reactions were then diluted and plated on blood agar plates. The percentage of the engulfed inoculum (at 15 minutes) that was killed was then calculated.

### Bacterial Uptake Assay

Bacterial uptake was determined using inside-out staining as previously described (Bhalla et al., 2020). PMNs were infected with GFP-expressing *S. pneumoniae* at a MOI of 2. Reactions were incubated rotating for the indicated times at 37°C. Cells were washed and resuspend in FACS buffer. To differentiate between associated vs. engulfed bacteria, the cells were stained with rabbit polyclonal anti-pneumococcal serotype 4 capsular antibodies (Cederlane) followed by a PE-conjugated secondary anti-Rabbit IgG antibody (12473981; Invitrogen). Flow cytometry was used to determine the percentage of PMNs that associated with bacteria (GFP^+^ PMNs). GFP^+^ PMNs were analyzed for a PE signal and the percentage of engulfed bacteria (GFP^+^/PE^-^) vs. extracellular bacteria (GFP^+^PE^+^) was determined. To assess the amounts of engulfed bacteria, we gated on GFP^+^/PE^-^ PMNs and measured the mean fluorescent intensity of GFP within that gate.

### Adoptive Transfer of PMNs

PMNs were adoptively transferred as previously described (Bhalla et al., 2020; Siwapornchai et al., 2020). Briefly, PMNs were isolated from the bone marrow of young and old naïve (unvaccinated), uninfected mice and resuspended in PBS. 2.5x10^6^ PMNs were then transferred into old PCV vaccinated mice by intraperitoneal (i.p) injection. This method allows delivery of PMNs into the circulation as we previously described (Siwapornchai et al., 2020). One hour following transfer, mice were infected i.t with 1x10^6^ Colony Forming Units (CFU) *S. pneumoniae* TIGR4. At 18 hours post infection, the mice were scored for clinical signs of disease. Mice were euthanized and the lung and blood collected and plated on blood agar plates for enumeration of bacterial CFU.

### Measurement of Extracellular DNA

PMNs were infected at a MOI of 2 with *S. pneumoniae* TIGR4 pre-opsonized with 3% naïve or heat inactivated immune sera for 40 minutes at 37°C. Control wells were mock infected with 3% sera only. Cells were then centrifuged to collect the supernatants. Supernatant samples were then stained for DNA with SYTOX Green (Invitrogen) and DNA was measured using a Biotek plate reader.

### Antibody ELISA

Antibody levels against heat-killed *S. pneumoniae* TIGR4 were measured by ELISA as previously described (Bou Ghanem et al., 2018; Tchalla et al., 2020; Bhalla et al., 2021).

### Antibody Binding to Bacterial Surfaces

WT and capsule deletion mutant (Δcps) *S. pneumoniae* TIGR4 bacteria were opsonized with 3% heat inactivated immune, immune, or naïve sera at 37°C for 30 minutes. Cells were washed, pelleted and stained for IgG antibodies with APC conjugated IgG (H+L) F(ab ft.) Goat anti-Mouse (17-4010-82; eBioscience). Flow cytometry was used to measure the MFI of antibody bound to the surface of bacteria.

### Complement Deposition Assay

WT *S. pneumoniae* TIGR4 bacteria were opsonized at 37°C for 30 minutes with 3% naïve, heat inactivated naïve, immune, or heat inactivated immune sera as indicated. Following opsonization, cells were washed, pelleted, and stained for complement with FITC conjugated Goat anti-mouse C3 (Catalogue number GC3-90F-Z; purchased from ICL). Flow cytometry was used to measure the MFI of complement bound to the surface of bacteria.

### Neutrophil Depletion

Neutrophils were depleted by intraperitoneal injection of 50ug of anti Ly6G antibody (clone IA8) or isotype IgG control (BD Pharmingen) following the timeline outlined in **Figure 1A** (Tchalla et al., 2020).

### Flow Cytometry

Fluorescence intensities were measured on a BD Fortessa and at least 20,000 events were analyzed using FlowJo.

### Statistics

Statistical analysis was performed using Prism 9 (Graph Pad). CFU data were log-transformed to normalize distribution. Bar graphs represent the mean values +/- SD. Significant differences were determined by Student’s t-test, one-way ANOVA followed by Dunnet’s or Tukey’s multiple comparisons test or 2-way ANOVA followed by Sidak’s multiple comparisons test as appropriate (indicated in the legends). Differences between fractions were determined by Fisher’s exact test. Survival analyses were performed using the log-rank (Mantel-Cox) test. All *p* values less than 0.05 were considered significant (as indicated by asterisks). * denotes p<0.05, ** denotes p<0.01, and *** denotes p<0.001.

## Results

### PMNs Are Required for Protection in PCV Vaccinated Young Hosts Following Bacterial Infection

PMNs are required for innate resistance of naïve hosts against *S. pneumoniae* infection, however, the role of neutrophils in anti-bacterial defense in vaccinated hosts is unclear. To determine the role of PMNs in response to *S. pneumoniae* in vaccinated hosts, young, C57BL/6 female mice were immunized with pneumococcal conjugate vaccine (PCV). Four weeks later, immunized mice were treated with isotype controls or anti-Ly6G Ab IA8 to deplete PMNs one day prior to and daily throughout the first 4 days following *S. pneumoniae* pulmonary infection (**Figure 1A**). Depletion of PMNs prior to intratracheal challenge with *S. pneumoniae* and throughout the course of infection resulted in loss of vaccine-mediated protection (**Figure 1**). While 100% of PMN sufficient vaccinated controls survived a challenge dose that is lethal in naïve mice, only 25% of PMN depleted vaccinated mice survived the infection (**Figure 1B**). As biological sex can influence immune responses, and our studies in old mice are limited to males due to availability, we tested the role of PMNs in young vaccinated male mice. Similar to what we found in females, PMN depletion of vaccinated young male mice resulted in a significant decrease in survival and loss of vaccine-mediated protection (**Figure S1**). These data show that PMNs are required for PCV-mediated protection against *S. pneumoniae* infection in young hosts.

### The Presence of Immune Sera Enhances Opsonophagocytic Killing in Vaccinated Young Hosts

**Figure 2:**
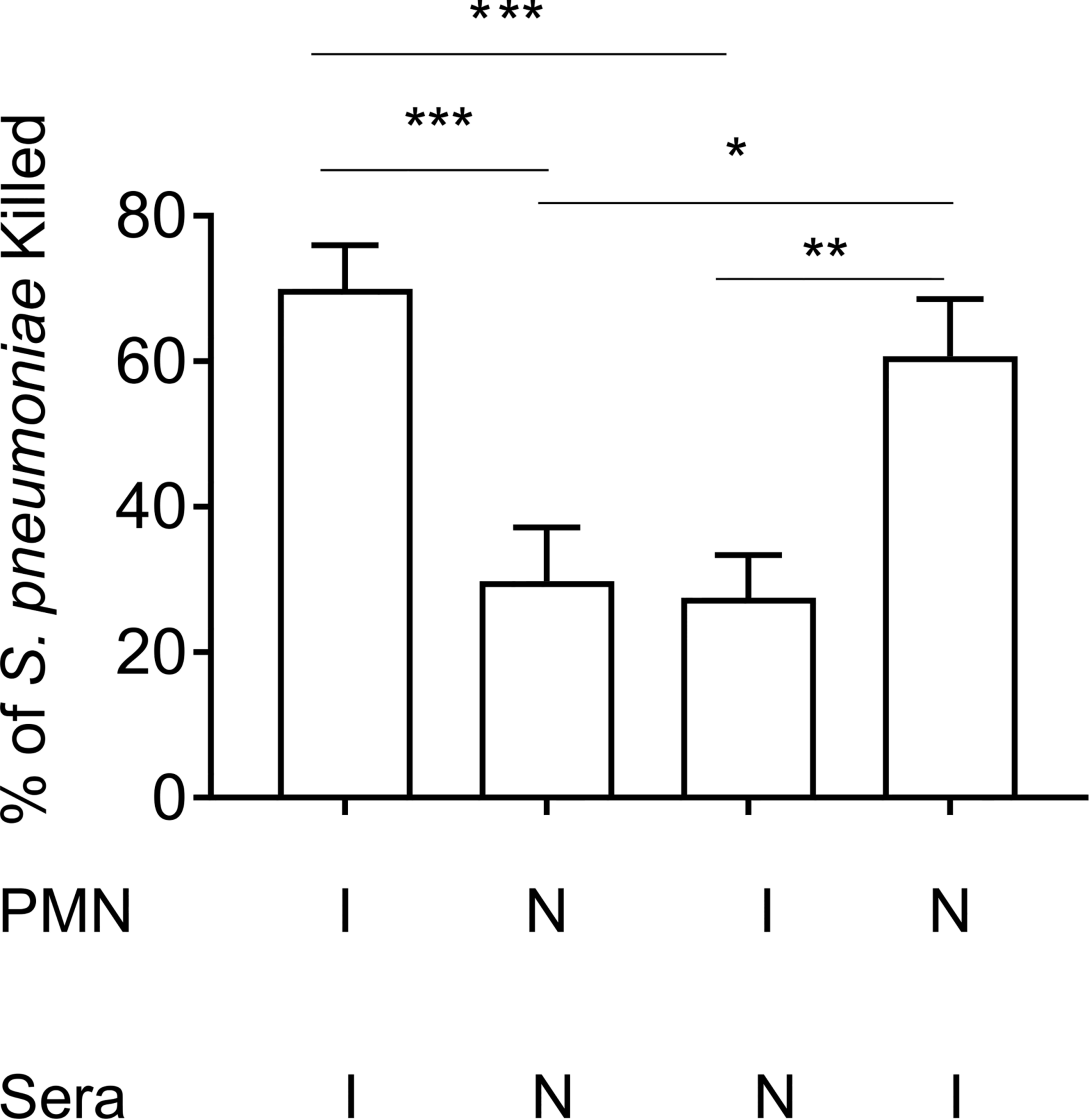
Enhanced opsonophagocytic bacterial killing by PMNs from vaccinated mice is dependent on opsonization by immune sera. PMNs were isolated from the bone marrow of young male C57BL/6 naïve (N) or Prevnar-13 immunized mice (I) and infected with *S. pneumoniae* pre-opsonized with either naïve or immune sera. The percentage of bacteria killed was then calculated with respect to a no PMN control under the same treatment conditions. Data shown are pooled from three separate experiments (n = 3 biological replicates) where each condition was tested in triplicate (n = 3 technical replicates) per experiment. *, denotes significance as calculated by One-way ANOVA followed by Tukey’s multiple comparisons test. * denotes p < 0.05, ** denotes p < 0.01, and *** denotes p < 0.001.

To investigate the role of PMN conferred protection in vaccinated hosts, we compared the ability of PMNs from immunized and naïve mice to kill *S. pneumoniae ex vivo* using established opsonophagocytic killing assays (Lysenko et al., 2007; Bou Ghanem et al., 2015). We found that PMNs from immunized mice killed *S. pneumoniae* significantly better than those isolated from naïve mice (**Figure 2**). This enhanced killing was a result of the presence of anti-pneumococcal antibodies in sera and not intrinsic to the PMNs themselves. When exposed to bacteria opsonized with naïve sera, the ability of PMNs from vaccinated mice to kill *S. pneumoniae* was comparable to that of naïve hosts (**Figure 2**). Similarly, the ability of PMNs from naïve mice to kill *S. pneumoniae* pre-opsonized with immune sera was significantly boosted (**Figure 2**). Sera isolated from immune mice contained significantly higher levels of IgG antibodies compared to naïve controls (**Figure S2A**). These antibodies bound to the surface of *S. pneumoniae* and were specific to the pneumococcal capsule (**Figure S2B**). These findings suggested that PMNs promote clearance of antibody opsonized bacteria. In fact, when we compared bacterial burdens in vaccinated mice following pulmonary challenge with *S. pneumoniae*, we found that the vaccinated PMN depleted hosts had a 100-fold higher bacterial burden in the lungs (**Figure 1C**) compared to vaccinated PMN sufficient controls. The vaccinated PMN depleted hosts also had systemic spread of the infection, where half of the mice became bacteremic compared to only 10% of the vaccinated isotype treated controls (**Figure 1D**). Overall, these findings suggest that following vaccination, PMNs act as effectors that promote clearance of antibody opsonized bacteria enhancing host resistance to infection.

### Anti-Pneumococcal Antibodies Enhance PMN Anti-Microbial Responses

**Figure 3:**
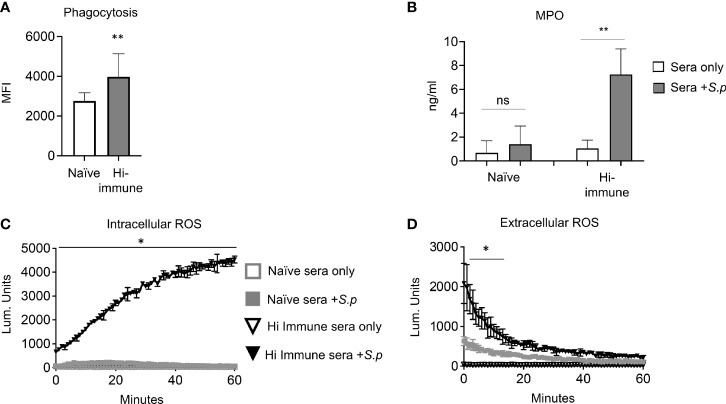
PMN anti-bacterial responses are enhanced in the presence of specific anti-pneumococcal antibodies. **(A)** PMNs were isolated from the bone marrow of young male C57BL/6 naïve mice and infected for 10 minutes with GFP tagged *S. pneumoniae* TIGR4 preopsonized with naïve or heat inactivated immune sera. The amount of GFP positive bacteria (MFI) inside each cell was determined *via* flow cytometry using inside-out staining. Data are pooled from three separate experiments where each condition was tested in triplicate (n = 3). Significant differences (*p < 0.05*, indicated by *) were determined by unpaired Student’s t test. **(B–D)** PMNs isolated from the bone marrow of young male C57BL/6 naïve mice were infected with *S. pneumoniae* pre-opsonized with naïve or heat inactivated (Hi-) immune sera or mock treated with sera alone as indicated. **(B)** The supernatants were t collected and myeloperoxidase (MPO) levels measured by ELISA. *, denotes significant difference compared to uninfected controls as determined by unpaired Student’s t test. Data are pooled from 3 separate experiments with n = 3 mice per group. **(C)** Production of intracellular ROS over time was measured by chemiluminescence of luminol. **(D)** Production of extracellular ROS over time was measured by chemiluminescence of isoluminol in the presence of HRP. **(C, D)** Data are representative of one of three separate experiments where each condition was tested in triplicate (n = 3 technical replicates) per experiment. Significant differences (*p < 0.05*, indicated by *) were determined by 2-way ANOVA followed by Sidak’s multiple comparisons test. * denotes p < 0.05, and ** denotes p < 0.01.

PMNs can kill bacteria through several antimicrobial functions. To determine the mechanisms by which immune sera enhances PMN activity, we analyzed several PMN functions in response to *S. pneumoniae* opsonized with naïve, or heat inactivated (Hi) immune sera. Heat inactivated immune sera was used to denature complement proteins and isolate the role of antibodies as an opsonin. We confirmed that heat inactivation of sera had no effect on antibody binding to the pneumococcal capsule when compared to full immune sera (**Figure S2B**). We also confirmed that heat inactivation of sera prevented complement deposition on the surface of bacteria (**Figure S2C**). To probe mechanisms, we assessed phagocytosis, degranulation and ROS production, which have all been shown to be important for the ability of PMNs to kill *S. pneumoniae* (Domon and Terao, 2021). We first measured phagocytosis of *S. pneumoniae* by PMNs using GFP-tagged bacteria and a flow-cytometry based assay we had previously established (Bhalla et al., 2020) and found that significantly more bacteria are taken in by PMNs when opsonized with Hi immune sera compared to when opsonized with naïve sera (**Figure 3B**). As the ~ 1.7-fold increase in the amount of engulfed bacteria did not fully account for the overall increase in killing capacity, we also assessed other antimicrobial activities. We measured the amount of Myeloperoxidase (MPO) released by PMNs into the supernatants in response to infection as a proxy for mobilization of primary granules (Armstrong et al., 2016; Miralda et al., 2020). We found that PMNs released 7-fold more MPO upon infection with *S. pneumoniae* opsonized with heat inactivated immune sera compared to bacteria opsonized with naïve sera (**Figure 3A**). Finally, we measured production of intracellular and extracellular reactive oxygen species (ROS) by PMNs using chemiluminescent assays. We found that when infected with *S. pneumoniae* opsonized with heat inactivated immune sera, both intracellular (**Figure 3C**) and extracellular (**Figure 3D**) ROS production by PMNs significantly increases when compared to infection by naïve sera opsonized bacteria. These data show that the presence of anti-pneumococcal antibodies in a PCV vaccinated host enhance PMN antimicrobial effector functions in response to *S. pneumoniae*.

### PCV Fails to Protect Old Mice Following *S. pneumoniae* Infection

**Figure 4:**
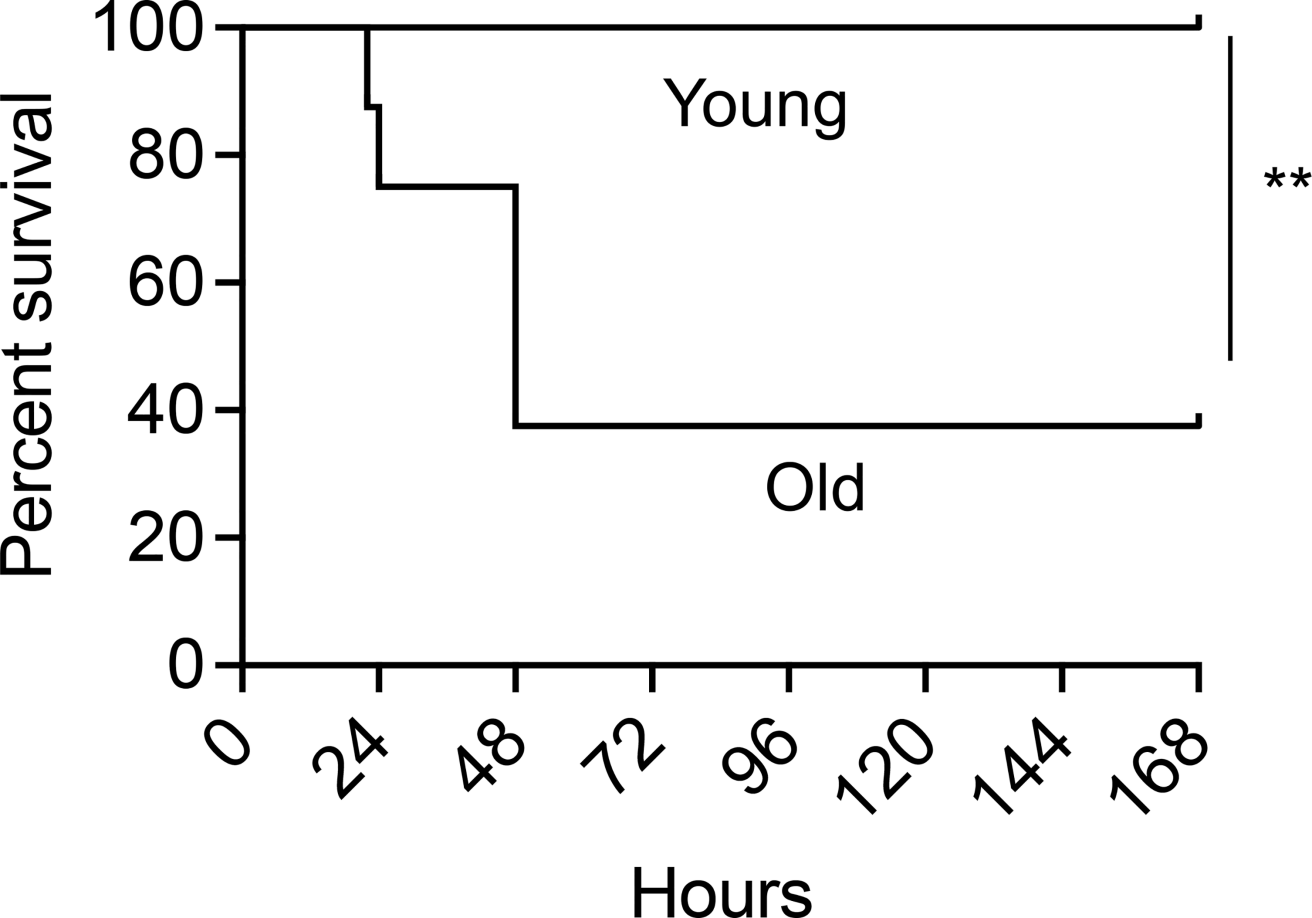
PCV administration fails to protect old mice against *S. pneumoniae* pulmonary infection. Young and old male C57BL/6 mice were vaccinated with Prevnar-13. Four weeks following vaccination, mice were infected intra-tracheally (i.t.) with 10^6^ CFU *S. pneumoniae* and monitored for survival. Data are pooled from 2 separate experiments with n = 8 mice per group. Significant differences were determined by Log-Rank (Mantel-Cox) test. ** denotes p < 0.01.

The findings above indicate that following vaccination, enhanced PMN antimicrobial function protects young hosts from pneumococcal infection, however, it is known that vaccine efficacy declines with age. To determine the efficacy of PCV vaccination using an aged murine model, young (2-3 months) and old (18-22 months) male C57BL/6 mice were vaccinated with PCV and four weeks following vaccination the mice were infected intratracheally with *S. pneumoniae* and monitored for survival. We found that while vaccinated young mice were fully protected, PCV immunization induced protection in less than half of the old mice, where only 37% survived the pulmonary challenge (**Figure 4**). These data show that PCV-mediated protection declines in aged hosts.

### Intrinsic PMN Function Declines With Age

**Figure 5:**
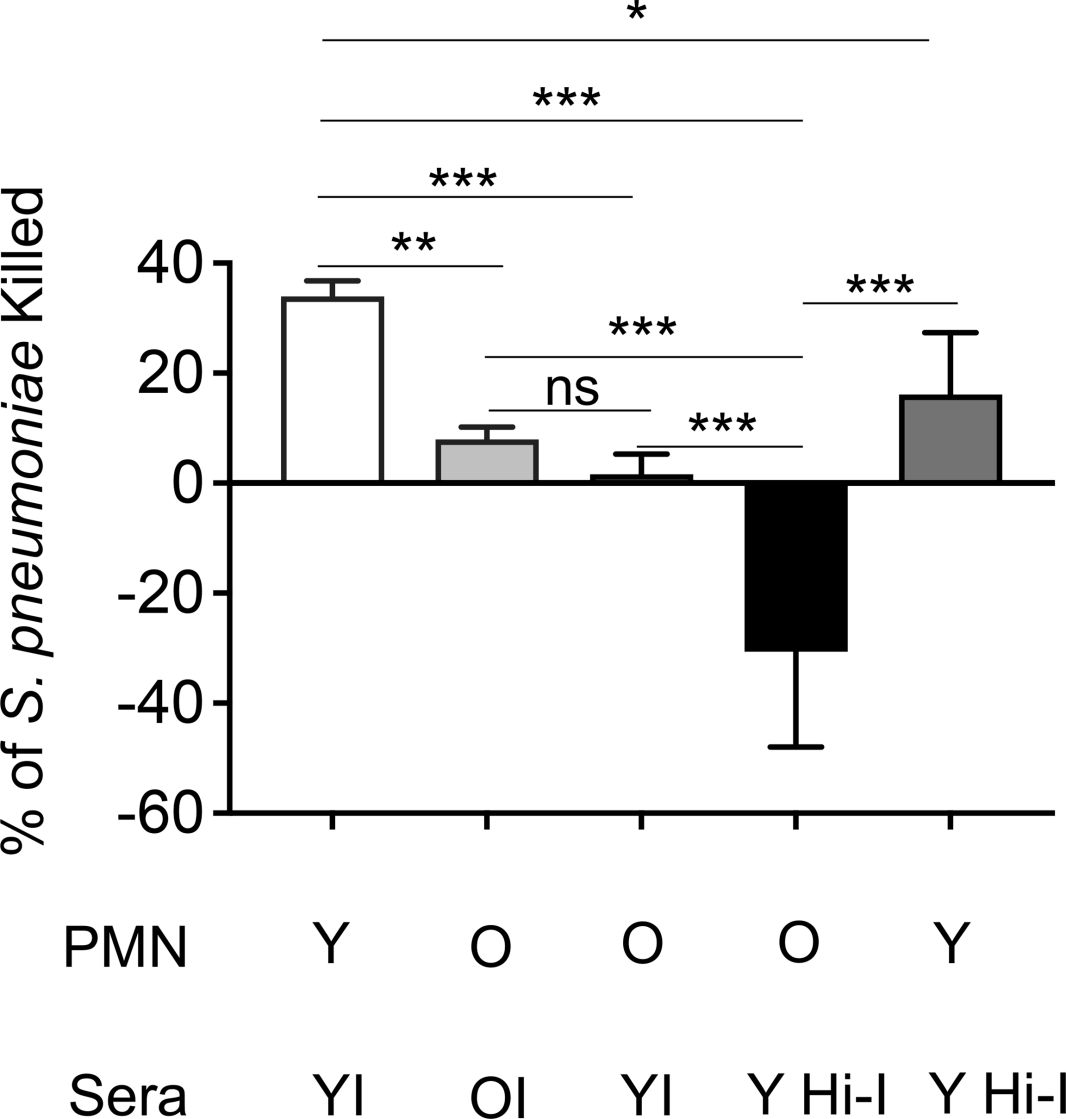
The intrinsic antibacterial function of PMNs declines with age. PMNs were isolated from the bone marrow of naïve young and old male C57BL/6 mice and infected with *S. pneumoniae* pre-opsonized with young immune (YI), old immune (OI), or young heat inactivated immune (Y Hi-I) sera. The percentage of bacteria killed was then calculated in comparison to a no PMN control under the same treatment conditions. Data are pooled from four separate experiments (n=4 biological replicates) where each condition was tested in triplicate (n=3 technical replicates) per experiment. *, denotes significant differences as determined by One-way ANOVA followed by Tukey’s multiple comparisons test. * denotes p < 0.05, ** denotes p < 0.01, and *** denotes p < 0.001. ns denotes not significant.

Given the decline in protection with age and the essential role of PMNs in vaccinated young hosts, we next looked at how PMN function changes with age upon vaccination. Using an opsonophagocytic killing assay, PMNs were isolated from young and old mice and exposed to *S. pneumoniae* opsonized with matching immune sera. We found that PMNs from vaccinated old mice kill pneumococci significantly worse than those isolated from young counterparts (**Figure 5**). It is well known that antibody levels and function following vaccination decline with age (Simell et al., 2011; Jackson et al., 2013). Therefore, to parse out the effect of the sera vs PMN intrinsic function, we mixed and matched sera and found that bacterial opsonization with immune sera from young controls failed to boost the antimicrobial activity of PMNs from old mice (**Figure 5**). This indicates that the decline in neutrophil function observed here is not solely due to the well-established age-related decline in anti-pneumococcal antibodies, but that there is an intrinsic decline in PMN function with age. Strikingly, when the immune sera from young mice were heat inactivated, PMNs from old mice completely failed to kill pneumococci and in fact bacterial growth occurred (**Figure 5**). This occurred only in the presence of PMNs from aged hosts as pre-opsonization with heat-inactivated immune sera still induced significant killing by PMNs from young mice (**Figure 5**), indicating there is an age-related decline in PMN killing of antibody opsonized *S. pneumoniae*. These data show that unlike in young hosts, immunization of old mice fails to boost PMN antimicrobial activity.

### Intracellular Killing of Engulfed *S. pneumoniae* Is Impaired With Age

**Figure 6:**
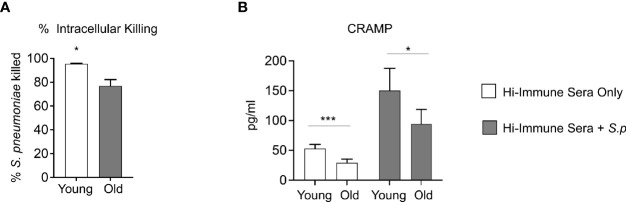
Aging impairs intracellular killing of engulfed *S. pneumoniae*. **(A)** PMNs were isolated from the bone marrow of naïve young and old male C57BL/6 mice and infected with *S. pneumoniae* pre-opsonized with young heat inactivated immune sera for 15 minutes at 37°C. Gentamicin (100μg/ml) was then added for 30 minutes to kill extracellular bacteria. PMNs were then washed and one set immediately plated on blood agar plates to determine the amounts of engulfed bacteria. The other sets of PMNs were incubated for 30 more minutes and then plated to enumerate remaining viable bacteria. The % of engulfed bacteria that was killed was then calculated. **(B)** PMNs from young and old naive mice were infected with *S. pneumoniae* pre-opsonized with young heat inactivated immune sera for 40 minutes. The cells were then lysed and assayed for CRAMP levels using ELISA. Data are pooled from **(A)** three separate experiments (n=3 biological replicates) where each condition was tested in triplicate (n=3 technical replicates) per experiment and **(B)** four separate experiments (n=4 biological replicates). *, denotes significant differences as determined by unpaired Student’s t test. * denotes p < 0.05, and *** denotes p < 0.001. ns denotes not significant.

To determine the mechanism of the intrinsic decline in PMN function observed with age, we compared the antimicrobial effector functions of PMNs isolated from young and old mice in response to *S. pneumoniae* opsonized with heat inactivated immune sera from young controls. We found that when comparing extracellular anti-microbial activities such as MPO release, extracellular ROS production, and extracellular DNA released as a marker of NETosis (Linnemann et al., 2020), there was no difference with age (**Figures S3A, C, E**). Additionally, we found no age-related difference in the amount of intracellular MPO levels or the amount of intracellular ROS produced (**Figures S3B, D**). As phagocytosis is required for the ability of PMNs to efficiently kill *S. pneumoniae* (Standish and Weiser, 2009) we next compared pneumococcal uptake by PMNs from young and old mice. We found no age-related difference in phagocytosis of bacteria opsonized with heat inactivated immune sera (**Figure S3F**). However, when we compared killing of engulfed bacteria using a gentamicin-protection assay we had previously established (Bhalla et al., 2020), we found a significant decline in the ability of PMNs from old mice to intracellularly kill *S. pneumoniae* opsonized with heat inactivated immune sera compared to PMNs from young mice (**Figure 6A**). An important mediator of pneumococcal intracellular killing by PMNs are antimicrobial enzymes and peptides packaged in intracellular granules within the cell (Yin and Heit, 2018). As we previously found no age-related difference in the intracellular concentration of the primary granule enzyme MPO (**Figure S3B**), we then analyzed the intracellular concentration of Cathelicidin-related antimicrobial peptide (CRAMP), a peptide that is able to kill pneumococci (Habets et al., 2012) and is found in PMN secondary granules (Borregaard et al., 2007). We found that at baseline, PMNs from old mice expressed significantly lower concentrations of CRAMP when compared to PMNs from young controls (**Figure 6B**). Following infection with *S. pneumoniae* opsonized with heat inactivated young immune sera, while both PMNs from young and old mice had increased levels of CRAMP, overall amounts were still significantly lower in aged hosts (**Figure 6B**). These data suggest that following antibody-mediated uptake, there is an age-related decline in the ability of PMNs to kill pneumococcus intracellularly, and this deficit in intracellular killing may be due to a decrease in intracellular CRAMP levels.

### Adoptive Transfer of PMNs From Young Mice Rescues the Age-Related Susceptibility of Vaccinated Old Mice to *S. pneumoniae* Infection

**Figure 7:**
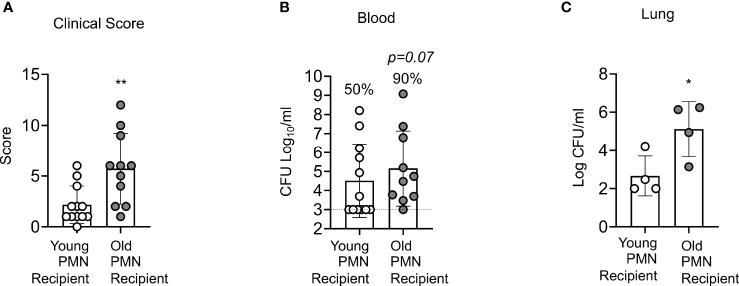
Adoptive transfer of PMNs from young mice at the time of challenge rescues the susceptibility of vaccinated old mice to *S. pneumoniae*. Old male C57BL/6 mice were vaccinated with Prevnar-13, four weeks post vaccination mice were adoptively transferred 2.5x10^6^ PMNs from either naïve young or naïve old mice. One hour post transfer, mice were infected i.t. with 1x10^6^ CFU of *S. pneumoniae* and clinical score **(A)** as well as bacterial numbers in the blood **(B)**, and lung **(C)** were determined 18 hours post infection. **(B)** Fractions indicate the percent of mice that were bacteremic. Asterisks indicate significant differences calculated by unpaired Student’s test **(A, C)** and Fisher’s exact test **(B)**. Data pooled from n=11 mice per group **(A, B)** or n=4 **(C)** mice per group are shown. * denotes p < 0.05, and ** denotes p < 0.01.

Aged mice are not protected following PCV vaccination and we have found that PMNs from aged mice fail to efficiently kill *S. pneumoniae* following antibody-mediated uptake. To determine if PMNs from young mice could boost the resistance of old PCV vaccinated mice to *S. pneumoniae* infection, we adoptively transferred 2.5x10^6^ PMNs isolated from the bone marrow of naïve young or old mice into four-week PCV vaccinated old mice. Following transfer of PMNs, mice were infected intratracheally with *S. pneumoniae*, and 18 hours post infection we analyzed the clinical score where a higher score corresponds to worse disease severity. We found that old PCV vaccinated mice that received PMNs from young controls had significantly lower clinical score compared to the old mice that received age matched PMNs (**Figure 7A**). The lower clinical score in the young PMN transfer group was accompanied by lower incidences of bacteremia with 50% of mice in this group becoming bacteremic compared with 90% of the mice in the aged matched transfer group (**Figure 7B**). Additionally, the old vaccinated mice that received PMNs from young controls also had significantly less bacterial burden in the lung compared to the age matched controls (**Figure 7C**). Taken together these data show that the presence of young, functional PMNs in an old vaccinated host reduces clinical disease presentation, and also reduces lung infection and systemic spread of the bacteria. These results indicate that enhancing PMN function in aged hosts improves anti-pneumococcal vaccine-mediated protection.

## Discussion

The role of PMNs in innate immunity and their interactions with *S. pneumoniae* have been well characterized (Kolaczkowska and Kubes, 2013; Simmons et al., 2021). However, many studies have been performed using un-opsonized bacteria, naïve sera (Standish and Weiser, 2009; Dalia et al., 2010; Bou Ghanem et al., 2015; Siwapornchai et al., 2020) or complement (Standish and Weiser, 2009; Bou Ghanem et al., 2017), while the interactions of PMNs with antibody-opsonized *S. pneumoniae* which have major implications on vaccine effectiveness are less explored (Fulop et al., 1985; Esposito et al., 1990; Butcher et al., 2001; Simell et al., 2011). This is of particular importance in hosts where immune responses to vaccination are sub-par as is the case in older individuals. The efficacy of pneumococcal vaccines is known to decline with age (Simell et al., 2011; Gonçalves et al., 2016; Matanock A et al., 2019) and while several aspects of the age-driven decline in adaptive immunity that result in reduced antibody production and function in response to immunization have been elucidated, the importance of PMNs in the decline of vaccine-mediated protection during aging remains under explored. In this study, we found that following PCV vaccination, PMNs are necessary for the protection of young hosts upon infection with *S. pneumoniae*. The binding of specific anti-capsular antibodies on the surface of the bacteria were shown to enhance several antimicrobial activities and overall pneumococcal killing by PMNs from young mice. However, aged mice were not protected by PCV vaccination and this decline in vaccine protection was associated with an age-related intrinsic decline in PMN function, specifically in antibody-mediated bacterial killing. Importantly, adoptive transfer of PMNs from young mice into aged hosts, rescued the ability of PCV to confer protection against infection. This work highlights both the importance of PMNs in protection of vaccinated hosts and that PMNs may be a potential target to boost vaccine-mediated protection in aged hosts that otherwise remain susceptible to *S. pneumoniae* infection.

*S. pneumoniae* have evolved several mechanisms to evade phagocytic uptake and clearance including the expression of capsule on their surface (Hyams et al., 2010; Shenoy and Orihuela, 2016). Therefore, opsonization of *Streptococcus pneumoniae* by complement and/or antibodies is important for bacterial uptake and clearance by PMNs (Kadioglu et al., 2008; Shenoy and Orihuela, 2016). Activation of complement receptors (CR) or Fc Receptors (FcR) on the PMN surface trigger distinct signaling pathways. It has been shown previously that activation of PMNs from young hosts and their resulting antimicrobial response is dependent on the receptor triggered (Kobayashi et al., 2002). When PMNs isolated from healthy, young, human donors were exposed to latex beads coated with IgG, serum complement, or both, phagocytosis and ROS production were affected based on the opsonin used (Kobayashi et al., 2002). IgG-coated beads induced faster phagocytosis and greater ROS production than beads coated with complement. In the case of ROS production the combined activation of both CRs and FcRs produced the highest amounts of ROS (Kobayashi et al., 2002). Additional studies using antibody or complement coated surfaces have shown that CRs alone do not initiate a strong ROS response but FcR-mediated activation or combined FcR and CR activation produce higher amounts of ROS (Zhou and Brown, 1994). PMN ROS response through the NADPH complex can also activate additional antimicrobial responses, such as NETosis and the production of proinflammatory cytokines (Nguyen et al., 2017). These data indicate that activation of antimicrobial effector functions by PMNs *via* CRs or FcRs proceed by distinct signaling pathways (Nguyen et al., 2017). In this study we used live bacteria to show that, similar to previous reports, PMNs isolated from young mice display increased antimicrobial effector functions when *S. pneumoniae* is opsonized with specific antibodies. When compared to opsonization with complement, opsonization of *S. pneumoniae* with antibodies alone resulted in a significant increase in overall pneumococcal killing and an increase in phagocytosis, ROS production, as well as MPO release.

Our work also shows an age-related decline in antibody-mediated bacterial killing by PMNs in mice immunized with the pneumococcal conjugate vaccine. This is in line with previous work in humans vaccinated with PCV, where PMNs isolated from the blood of elderly donors displayed a significant decline in opsonophagocytic killing when compared to PMNs isolated from young donors (Simell et al., 2011). This decline in opsonophagocytic killing with age was also accompanied by a significant decline in the opsonic titer of antibodies to multiple pneumococcal serotypes and there was an increase in the amounts of antibodies needed for functional bacterial killing to occur, however, complement activity was found to be higher with age (Simell et al., 2011). We showed here that when PMNs isolated from aged mice are exposed to *S. pneumoniae* opsonized with sera from immunized young donors, there was an age-related decline in pneumococcal killing. This impairment was even more pronounced when complement was deactivated, despite the presence of functional antibodies, suggesting that there is an intrinsic decline in select FcR-mediated antimicrobial responses with age. Indeed, pneumococci pre-opsonized with heat-inactivated sera were able to replicate in the presence of PMNs from aged mice. This is in line with our previous findings using these opsonophagocytic killing assays, where when PMNs fail to kill bacteria, we observe bacterial growth instead (Siwapornchai et al., 2020). It is known that *S. pneumoniae* express several exoglycosidases that can cleave terminal sugars off of glycoconjugates (King et al., 2006) and use those for growth (Burnaugh et al., 2008). Therefore, it is possible that *S. pneumoniae* are using factors expressed or released by PMNs such as glycoconjugates as a nutrient source, aiding bacterial replication.

We sought to determine the reason for this age-related decline, and found that in aged mice, following antibody-mediated uptake, PMNs have a decline in intracellular pneumococcal killing. To explore the mechanisms of this age-related decline in antibody-mediated intracellular killing we analyzed the intracellular levels of antimicrobial peptide CRAMP, a component of PMN secondary granules (Borregaard et al., 2007). In PMNs, secondary (also known as specific) granules, are released prior to the release of primary granules and play a role in the killing of pathogens (Yin and Heit, 2018). *S. pneumoniae* were shown to be susceptible to CRAMP and the human homologue LL-37 and clinical isolates of several different serotypes are killed upon exposure to LL-37 *in vitro* (Habets et al., 2012). CRAMP deficiency in CRAMP knockout mice was also shown to increase mortality in mice with pneumococcal meningitis (Merres et al., 2014). These data indicate that CRAMP is an antimicrobial product that is important for killing of *S. pneumoniae.* Our study shows that with age there is a decline in the intracellular levels of the antimicrobial peptide CRAMP in PMNs. This decline was both in resting PMNs and in response to infection suggesting less of this antimicrobial product is pre-made within PMNs and production of new CRAMP in response to infection is also impaired with aging. Interestingly, aged mice were also reported to display a decline in CRAMP expression in the epithelium of the upper respiratory tract, which was associated with a decline in pneumococcal clearance (Krone et al., 2013). Reduction in CRAMP abundance may partially account for the age-related decline in intracellular killing of bacteria by PMNs; however, as CRAMP production is reduced but not abolished, additional factors such as overall levels of granules, composition of the granular components, and their trafficking towards the bacteria containing phagosome in PMNs from aged hosts may also play a role and need to be investigated in the future.

With age there is a functional decline in antibody opsonic capacity and a decline in the number of antibodies produced in response to vaccination (Simell et al., 2011; Jackson et al., 2013). However, we found that introduction of PMNs from young hosts to aged vaccinated hosts was able to enhance host protection, despite the well-established decline in antibody number and function. These data indicate that in an immune aged host, despite a decline in adaptive immunity, the presence of functional PMNs is critical for protection against pneumococcal infection. This further suggests that the age-driven decline in PMN function also impairs vaccine-mediated protection against *Streptococcus pneumoniae*. As PMNs are crucial for host defense against a plethora of pathogens and their overall anti-microbial function declines with age (Butcher et al., 2000; Simmons et al., 2021), our findings have far-reaching implications to other infections in the elderly. Therefore, targeting PMN responses may be a potential future avenue for boosting overall vaccine efficacy in aged hosts.

## Data Availability Statement

The original contributions presented in the study are included in the article/**Supplementary Material**. Further inquiries can be directed to the corresponding author.

## Ethics Statement

The animal study was reviewed and approved by University at Buffalo Institutional Animal Care and Use Committee.

## Author Contributions

SS conducted research, analyzed data and wrote paper. ET and MB conducted research and analyzed data. ENBG designed research, wrote the paper and had responsibility for final content. All authors read and approved the final manuscript.

## Funding

This work supported by National Institute of Health grant R01AG068568-01A1 to ENBG. This work was also supported by American Heart Association Grant number 827322 to MB.

## Author Disclaimer

The content is solely the responsibility of the authors and does not necessarily represent the official views of the National Institutes of Health.

## Conflict of Interest

The authors declare that the research was conducted in the absence of any commercial or financial relationships that could be construed as a potential conflict of interest.

## Publisher’s Note

All claims expressed in this article are solely those of the authors and do not necessarily represent those of their affiliated organizations, or those of the publisher, the editors and the reviewers. Any product that may be evaluated in this article, or claim that may be made by its manufacturer, is not guaranteed or endorsed by the publisher.

## Supplementary Material

Supplementary Figure 1 PMNs are required for protection of PCV immunized young male hosts at the time of bacterial challenge. Young (2 months old) C57BL/6 male mice were mock treated (naïve) or administered 50μl of Prevnar-13 *via* intramuscular injections to the hind legs (vaccinated). Four weeks following vaccination mice were challenged i.t. with 1x10^6^ CFU *S. pneumoniae* TIGR4. To deplete PMNs prior to infection, mice were injected i.p. with anti-Ly6G antibodies (IA8) or isotype control at days -1, 0, and +1 to +4 with respect to infection as outlined in **Figure 1A**. Mice were then monitored for survival. Data were pooled from two experiments with 6 mice per group. Significant differences were determined using the Log-Rank (Mantel-Cox) test. ** denotes p<0.05, ** denotes p<0.01, and *** denotes p<0.001.*

Supplementary Figure 2 Production of anti-pneumococcal IgG in the sera following PCV administration. **(A)** Young C57BL/6 mice were mock treated (naïve) or vaccinated with Prevnar-13 and blood was collected at two and four weeks following vaccination. IgG levels against heat-killed *S. pneumoniae* TIGR4 were measured by ELISA. Antibody (Ab) units were calculated based on a hyperimmune standard included in each ELISA. Asterisks indicate significant differences with respect to vaccinated mice as determined by unpaired Student t-test. Data were pooled from two separate experiments with n=6 mice per group and presented as means +/- SD. **(B)** Sera was collected from four-week immunized or naïve male mice as indicated and used to opsonize *S. pneumoniae* TIGR4 (WT) or a capsular deletion mutant (*Δcps*) for 30 minutes. The cells were then washed and stained with fluorescently-labeled anti-mouse IgG. The amount (mean fluorescent intensity or MFI) of Abs bound to the bacterial surface was determined by flow cytometry. Representative data from one of three separate experiments (n=3 biological replicates) are shown where each condition was tested in triplicate (n=3 technical replicates) per experiment. *, denotes significant differences from WT bacteria opsonized with immune sera by One-way ANOVA followed by Dunnett’s multiple comparisons test. **(C)** Sera was collected from PCV immunized or naïve mice and heat inactivated at 56°C for 40 minutes as indicated. *S.pneumoniae* TIGR4 was opsonized with the sera for 30 minutes. Cells were labeled with fluorescently tagged anti-mouse C3 antibody and the amount (MFI) of antibody bound to complement was determined by flow cytometry. Data are pooled from n=3 separate experiments with each condition tested in triplicate (n=3). *, indicates significant difference by One-way ANOVA followed by Dunnett’s multiple comparisons test. ** denotes p<0.05, ** denotes p<0.01, and *** denotes p<0.001.*

Supplementary Figure 3 Aging does not affect production of ROS, MPO, release of extracellular DNA or bacterial uptake by PMNs in response to antibody-opsonized *S. pneumoniae.* PMNs were isolated from the bone marrow of naïve young and old male C57BL/6 mice and infected with *S. pneumoniae* pre-opsonized with heat inactivated immune sera from young mice (+*S.p*) or mock infected with heat inactivated immune sera alone (uninfected) as indicated. **(A, B)** Forty minutes post-infection, MPO concentrations present in the pellets or released in the supernatants were measured using ELISA. Data are pooled from three separate experiments. **(C)** The levels of extracellular DNA released in the supernatants were also measured by SYTOX Green. Data are pooled from three separate experiments (n=3 biological replicates per group). **(D, E)** Production of intracellular and extracellular ROS over time following infection was measured by chemiluminescence of luminol and isoluminol plus HRP respectively. Data are representative of one of three separate experiments where each condition was tested in triplicate (n=3 technical replicates) per experiment. **(F)** PMNs were infected for 15 minutes with GFP tagged *S. pneumoniae* pre-opsonized with heat-inactivated immune sera. The amount (MFI) of intracellular bacteria was determined be flow cytometry using inside-out staining. Data are pooled from three separate experiments (n=3 biological replicates per group).
