# Supplementary figures and images for: The Age-Driven Decline in Neutrophil Function Contributes to the Reduced Efficacy of the Pneumococcal Conjugate Vaccine in Old Hosts
Source: Front Cell Infect Microbiol. 2022 Mar 23;12:849224. doi: 10.3389/fcimb.2022.849224 (PMC8984502; doi:10.3389/fcimb.2022.849224)

Fig. S1

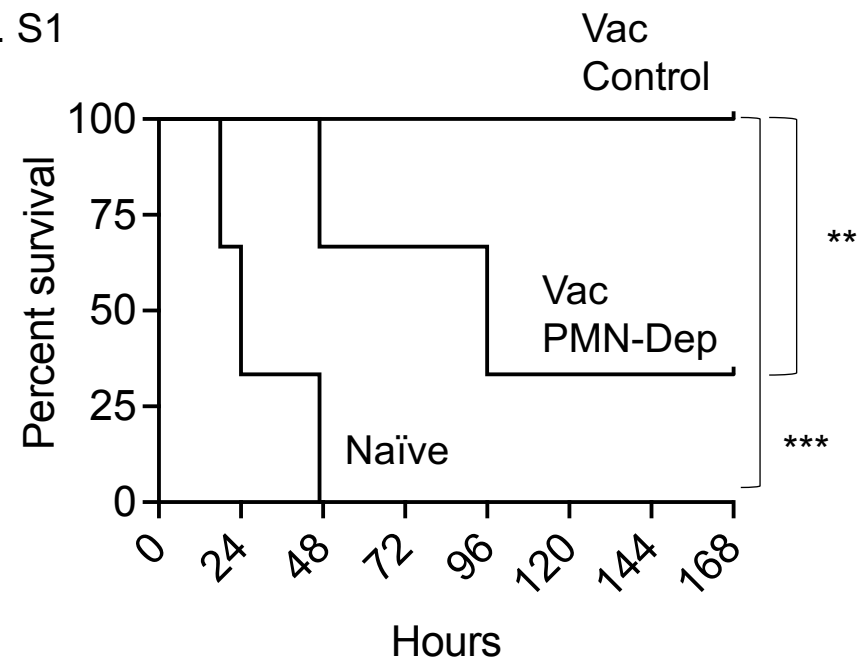

Fig. S2

A.

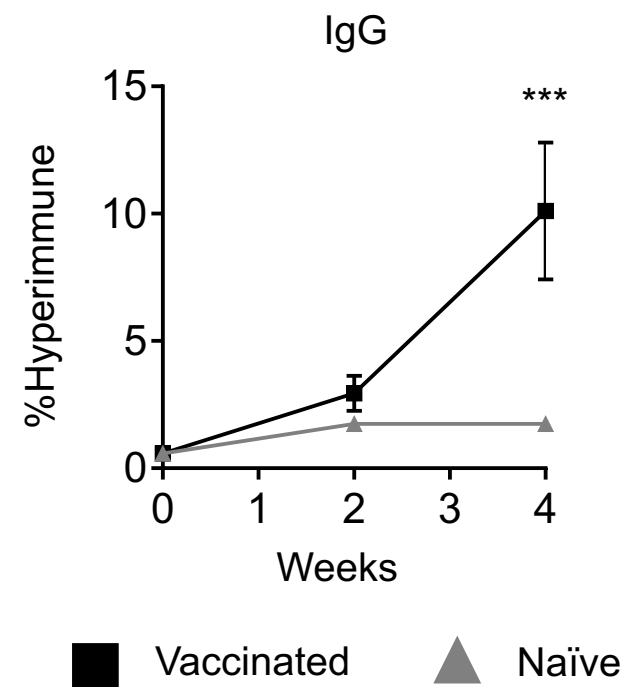

B.

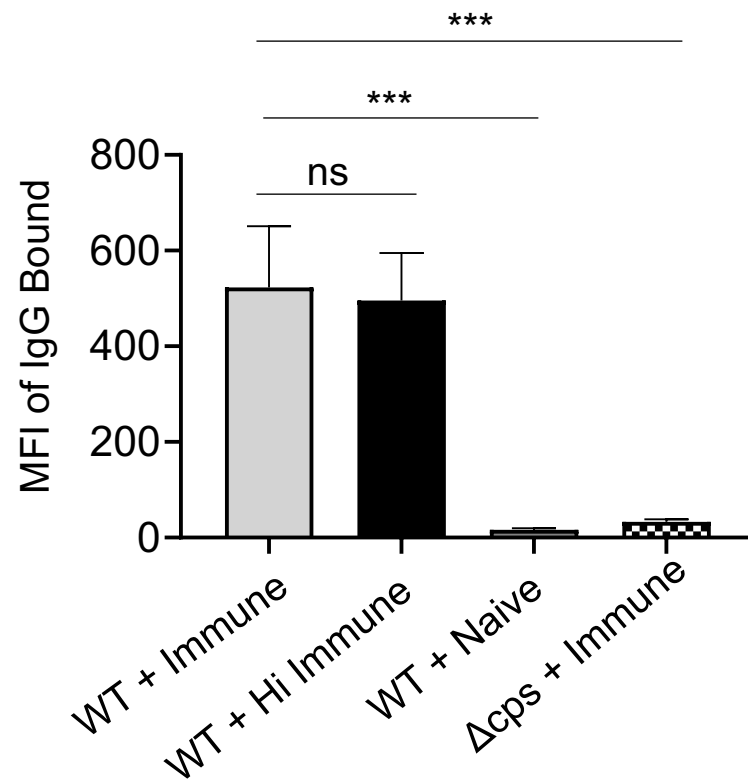

C.

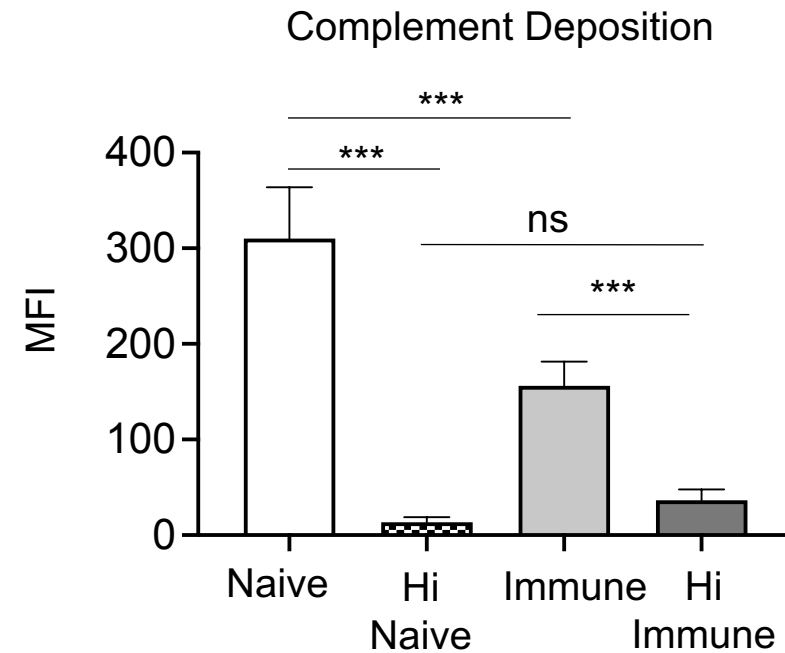

Fig. S3

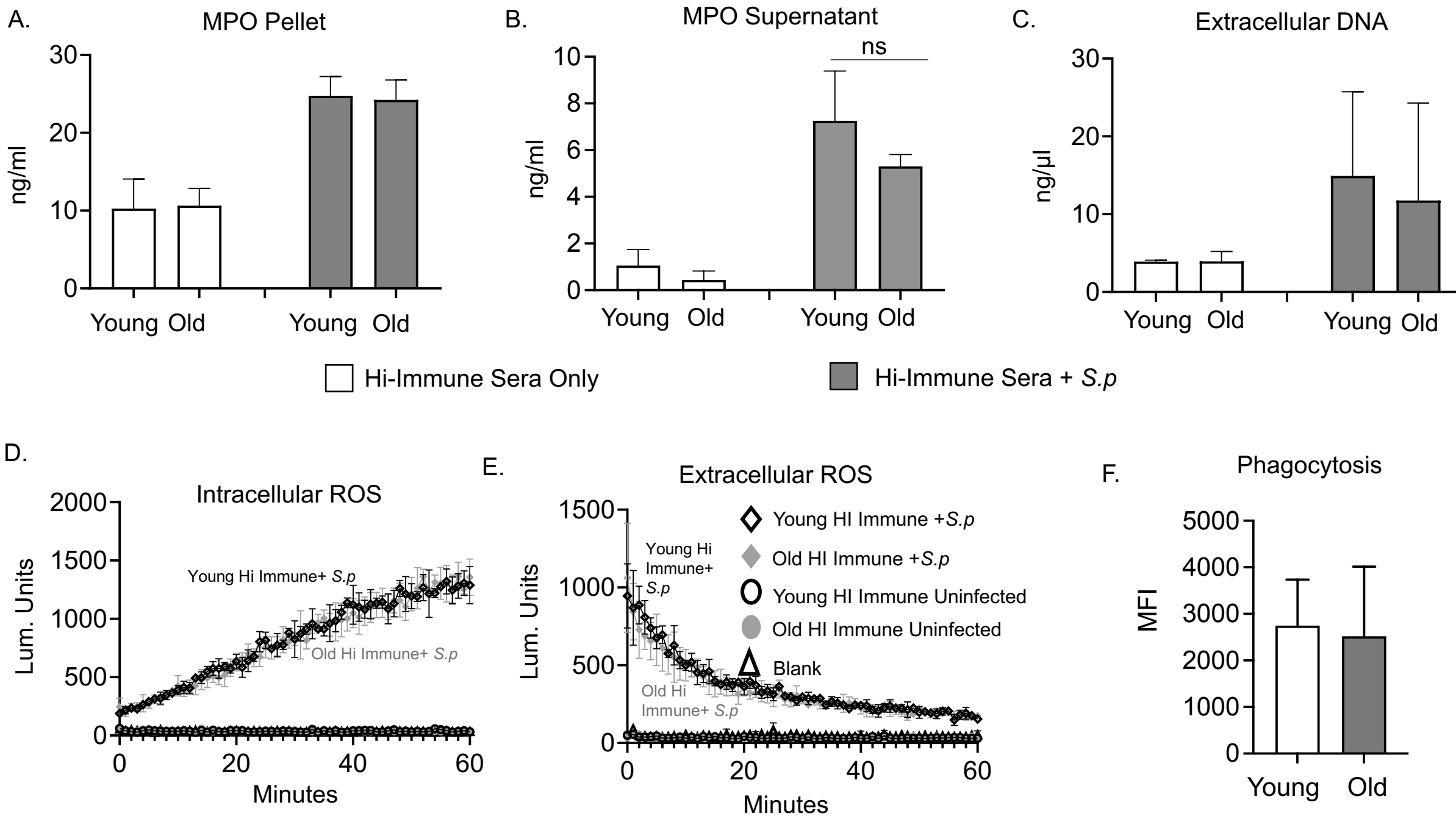

Supplement: Supplementary Figure 1 — PMNs are required for protection of PCV immunized young male hosts at the time of bacterial challenge. Young (2 months old) C57BL/6 male mice were mock treated (naïve) or administered 50μl of Prevnar-13 via intramuscular injections to the hind legs (vaccinated). Four weeks following vaccination mice were challenged i.t. with 1x10^6^ CFU S. pneumoniae TIGR4. To deplete PMNs prior to infection, mice were injected i.p. with anti-Ly6G antibodies (IA8) or isotype control at days -1, 0, and +1 to +4 with respect to infection as outlined in Figure 1A . Mice were then monitored for survival. Data were pooled from two experiments with 6 mice per group. Significant differences were determined using the Log-Rank (Mantel-Cox) test. * denotes p<0.05, ** denotes p<0.01, and *** denotes p<0.001. [file DataSheet_1.pdf]
